# Household and child correlates of child trafficking indicators in Eastern Sierra Leone

**DOI:** 10.1371/journal.pone.0347898

**Published:** 2026-10-05

**Authors:** Liu Liu, David Okech, Anna Cody

**Affiliations:** Center on Human Trafficking Research & Outreach, School of Social Work, University of Georgia, Athens, Georgia, United States of America; IFPRI: International Food Policy Research Institute, UNITED STATES OF AMERICA

## Abstract

Child trafficking indicators remain highly prevalent in Sierra Leone’s Eastern Province, yet less is known about whether household-level correlates are robust when child-level variation within households is considered. This study examined household and child correlates of child trafficking indicators using baseline survey data from 2,429 households in Kailahun, Kenema, and Kono districts, including 5,999 rostered children aged 5–17. Child trafficking indicators were constructed from the survey’s operational definition, including hazardous sectors, hazardous work conditions, and force, fraud, coercion, or restriction indicators. We estimated three complementary models: a household-level logistic model estimating associations with whether any child in the household had trafficking indicators, a grouped-binomial household model estimating associations with the number of affected children out of all rostered children aged 5–17, and a child-level logistic model estimating associations with each child’s trafficking indicator status. Primary models used enumeration-area-clustered standard errors, with random-intercept models as sensitivity analyses. Overall, 49.2% of households had at least one child with trafficking indicators, and 33.8% of children had one or more trafficking indicators. Subjective economic strain was not a robust positive correlate in the primary household-level, grouped-binomial, or child-level models. In contrast, objective material wealth, school enrollment vulnerability, orphanhood, child age, and district context showed more consistent associations. In the primary household model, material wealth, having any child not enrolled in formal school, and having any orphan child were associated with higher odds of household trafficking indicators. In child-level models, older age and orphanhood were associated with higher odds, while female sex and formal school enrollment were associated with lower odds. Kono showed elevated trafficking indicators relative to Kenema in grouped-binomial and child-level models. Findings suggest that child trafficking indicators in Eastern Sierra Leone are patterned by household, child, and district context, and are not fully explained by subjective economic strain. Prevention strategies should move beyond a simple poverty-only framework and integrate school access, support for orphaned and out-of-school children, and place-based responses in higher-risk districts.

## Introduction

Child trafficking, broadly understood as the recruitment, transfer, harboring, or receipt of a child for exploitative labor or commercial sexual exploitation, remains a major child protection and development concern globally and in Sierra Leone [1–3]. In Sierra Leone’s Eastern Province, prior population-based work has documented high levels of trafficking indicators among children aged 5–17, underscoring the need for context-specific evidence on the household, child, and district conditions associated with trafficking vulnerability [4]. Such evidence is especially important because trafficking indicators in this setting are not limited to a single form of exploitation. They may include hazardous sectors, hazardous work conditions, and force, fraud, coercion, or restriction-related indicators. Understanding how these indicators are patterned across households and children can inform prevention strategies that are better aligned with local economic and social realities.

Research on child trafficking and child labor in Sierra Leone and West Africa points to a set of overlapping vulnerabilities. Poverty, insecure livelihoods, limited schooling, household caregiving disruption, and the misuse of informal fostering or kinship care arrangements can create conditions in which children become exposed to exploitative work or deceptive recruitment [1,4–6]. Recruiters may promise schooling, apprenticeship, or employment opportunities, while actual outcomes may include domestic servitude, street vending, mining, portering, fishing, or other hazardous labor [1,5,7–9]. These dynamics suggest that child trafficking indicators are shaped by both household-level vulnerability and local labor-market context.

Economic conditions are central to this literature, but economic vulnerability is multidimensional. One dimension is objective material living standards, often measured in low-resource settings using asset-based wealth indices derived from household assets and housing or utility characteristics [10–12]. Such indices are widely used when income or consumption data are unavailable or unstable, and they capture durable features of household welfare. A second dimension is subjective economic strain, or the household’s perceived difficulty meeting basic needs. The Family Stress Model distinguishes objective economic hardship from subjective economic pressure and argues that perceived strain can affect caregiver well-being, household decision-making, and child outcomes [13–16]. In trafficking research, this distinction is important because two households with similar assets may differ in debt, obligations, expectations, coping capacity, or exposure to shocks. Conversely, households with more assets may also be more connected to local economic activities where children’s labor becomes visible or demanded.

Schooling is another important dimension of vulnerability. School enrollment may reduce exposure to hazardous work by providing supervision, structure, institutional visibility, and access to referral pathways. When schooling is interrupted by fees, distance, caregiving disruption, labor demand, or social norms, children may become more available for work or migration pathways that can be exploited [17–19]. In Sierra Leone, prior studies have documented that promises of education are commonly used in recruitment narratives, and that irregular or absent school participation can increase children’s exposure to exploitative arrangements [1,4,5]. Therefore, school enrollment should be examined not only as an educational outcome but also as a child protection marker.

Caregiving structure and household composition may also shape vulnerability. Orphaned children or children in disrupted caregiving arrangements may experience reduced supervision, greater pressure to contribute economically, placement in extended households, or reliance on informal arrangements that can be manipulated by recruiters. Household size and composition may matter as well. Larger households may face resource dilution when more dependents stretch limited resources, but extended households can also provide additional caregivers or income earners [20–22]. These mixed possibilities suggest that household composition should be modeled carefully and that household-level outcomes should not be assumed to represent the same process as child-level risk.

A related methodological challenge is that child trafficking indicators are often summarized at the household level. A household-level indicator, such as whether any child in the household has trafficking indicators, is useful for program targeting because it identifies households where intervention may be needed. However, this approach can obscure variation among children within the same household. A household with one affected child and a household with several affected children are both coded positive under an any-child rule. Similarly, associations observed at the household level may differ from associations observed at the child level. Addressing this issue requires analyses that connect household-level vulnerability with child-level outcomes and test whether conclusions are robust across levels of aggregation.

District context is also likely to be important in Sierra Leone’s Eastern Province. Kailahun, Kenema, and Kono differ in economic structure, mobility patterns, services, and local labor opportunities. Prior reports and studies have highlighted mining-related economies and informal labor markets in parts of the Eastern Province, particularly Kono, as potential contexts in which children may become exposed to hazardous or exploitative work [23–25]. Although household-level characteristics are important, district-level differences may indicate broader structural conditions that shape children’s exposure to trafficking indicators.

The present study examines economic and social correlates of child trafficking indicators among households and children in Kailahun, Kenema, and Kono districts of Sierra Leone’s Eastern Province. This manuscript differs from prior reports from the broader project by focusing specifically on the linked household- and child-level structure of the baseline survey data and by evaluating whether associations are consistent across different ways of defining trafficking indicators at the household and child levels. We make three contributions. First, we examine subjective economic strain and objective material wealth as distinct household economic measures rather than treating poverty as a single construct. Second, we examine child and household vulnerability markers, including formal school enrollment, orphanhood, child age, sex, household size, and district context. Third, we address the aggregation of child-level trafficking indicators into household-level outcomes by estimating three complementary outcome specifications: a policy-relevant household binary outcome indicating whether any child in the household had trafficking indicators, a grouped-binomial household outcome representing the number of affected children out of all rostered children aged 5–17, and a child-level binary outcome. This design allows us to evaluate whether findings are consistent across household-level and child-level definitions of trafficking indicators while accounting for the multistage sampling structure.

## Method

### Data sources and sampling

We used data from a baseline household survey conducted as part of a multi-level implementation project to inform counter-trafficking prevention and response efforts in Sierra Leone’s Eastern Province. The baseline survey used a multistage cluster sampling design in three districts: Kailahun, Kenema and Kono. In the first stage, enumeration areas were selected within districts. In the second stage, households were randomly sampled within selected enumeration areas. Enumeration areas were treated as the primary sampling units in all design-adjusted analyses.

The original household survey included 2,990 interviewed households. For the present analysis, we restricted the analytic household sample to households with at least one rostered child aged 5–17 and a defined child trafficking indicator. This restriction was necessary because the primary household-level outcome was defined by whether any child aged 5–17 in the household had trafficking indicators. Households without rostered children aged 5–17 were therefore not part of the outcome risk set. The final household-level analytic sample included 2,429 households. These households included 5,999 rostered children aged 5–17. For analyses that used child-level outcomes, the analytic sample included the same rostered children aged 5–17, with model-specific complete-case sample sizes reported in the relevant tables.

Interviews were conducted face to face with household heads or other knowledgeable adult respondents aged 18 years or older. Trained interviewers administered the structured survey orally and recorded responses during the interview. No children were interviewed directly. Information about children’s work, schooling, household membership, and trafficking-relevant experiences was reported by adult household respondents. Model-specific analytic sample sizes are summarized in S1 Table.

### Ethical considerations

Data collection took place between December 2019 and August 2021. Ethical approval was obtained from the Government of Sierra Leone Office of the Sierra Leone Ethics and Scientific Review Committee, the ethics committee at Makerere University (Ref No. 2019−060), the University of Liverpool Research Ethics Committee (Reference 5622), and the University of Georgia Institutional Review Board (PROJECT00000941, PROJECT00003415). Where applicable, protocol amendments and extensions were approved and covered the full period of study activities and data access.

All respondents received an information sheet describing the study aims, procedures, potential risks and benefits, and confidentiality protections. Interviewers obtained written informed consent by requiring a signature or thumbprint from participants with limited literacy. Participation was voluntary, and respondents could refuse to answer any question or stop the interview at any time without consequence. No children were interviewed directly. Information about children’s work and trafficking- related experiences was reported by adult caregivers. Interviewers were trained in child protection and referral procedures, and they provided contact information for local support services when needed. The de-identified baseline analytic dataset was accessed for research purposes from October 1, 2021 to February 28, 2022.

### Measures

#### Child trafficking indicator.

Child trafficking was operationalized using child-level survey indicators from the household roster and child work modules. The classification followed the baseline survey’s operational definition rather than a legal adjudication of trafficking victimization. A rostered child aged 5–17 was classified as having trafficking indicators if the adult respondent reported any “yes” response to one or more predefined trafficking-relevant items. The child trafficking indicator was developed for the baseline survey using the study’s conceptual, statistical, and operational definitions of child trafficking, and the survey instrument was pretested and piloted before full implementation [4]. The classification was designed as a criterion-based operational measure that captures multiple trafficking-relevant conditions, including hazardous sectors, hazardous work conditions, and force, fraud, coercion, or restriction indicators. Its content validity is supported by alignment with the study’s operational definition and by transparent reporting of the specific survey items used to classify children.

The operational definition included three groups of indicators. First, children were classified as positive if they were reported to have engaged in selected hazardous or exploitative sectors, including mining or quarrying, fishing, portering or casual labor, sex work, manufacturing, or construction. Second, children were classified as positive if they were reported to have performed work away from home under hazardous conditions, including carrying heavy loads, using dangerous tools or operating heavy machinery, exposure to dust, fumes, or gas, exposure to extreme cold, heat, or humidity, or exposure to loud noise or vibration. Third, children were classified as positive if the respondent reported force, fraud, coercion, or restriction indicators, including being forced to work for someone outside the household, being forced to work to repay a debt, working outside the home for little or no wages, performing work that was not agreed upon, being forced or made to beg, performing illegal or immoral work, being forced or made to work to pay school fees, or not being allowed to leave or contact parents.

We constructed three outcome variables from this child-level classification. The first was a child-level binary indicator coded 1 if the child had any trafficking indicator and 0 otherwise. The second was a household-level binary indicator coded 1 if at least one rostered child aged 5–17 in the household had any trafficking indicator and 0 otherwise. This household-level binary indicator was retained as the primary policy-relevant household vulnerability outcome. The third was a household-level count/proportion outcome: the number of children with trafficking indicators out of the total number of rostered children aged 5–17 in the household. This alternative household outcome was used to evaluate whether results were robust to less aggregated outcome definitions.

#### Subjective economic strain.

Subjective economic strain was measured using six household welfare items. Respondents were asked to indicate their agreement with statements about household economic well-being and resource adequacy on a 5-point scale ranging from 1 = strongly agree to 5 = strongly disagree. The items asked whether the household was well off by local standards, whether the household found it difficult to live on its current income, and whether the household generally had enough food, money for school fees, clothing, and medicine.

Items were keyed so that higher scores indicated greater perceived economic strain. For the item about difficulty living on current income, stronger agreement indicated greater strain, so this item was reverse-coded. For the well-off item and the four resource-adequacy items, stronger disagreement indicated greater strain, so these items were retained in their original direction. The six keyed items were averaged to create a household-level economic strain scale. Households with at least four observed items were assigned the mean of the available keyed items. The resulting scale was standardized for regression analyses so that coefficients represent the association with a one-standard-deviation difference in perceived economic strain.

The six-item scale showed good internal consistency in the analytic sample. In sensitivity checks, results were similar when a five-item version excluding the well-off item was used. Because the six-item scale had good reliability and represented both perceived relative standing and concrete resource adequacy, it was retained as the primary subjective economic strain measure. Scale reliability and item-level diagnostics are reported in S2 Table.

#### Objective material wealth.

Objective material wealth was measured using the asset-based household wealth score available in the household analytic file. The score was derived from household assets and housing or utility characteristics collected in the baseline survey, oriented so that higher values reflected greater material advantage, and standardized within the analytic household sample. Thus, regression coefficients for wealth represent the association with a one-standard-deviation difference in objective material wealth.

Subjective economic strain and objective material wealth were treated as related but distinct dimensions of household economic conditions. Subjective strain captures perceived difficulty meeting household needs, whereas the wealth score captures observed material living standards. Including both measures allowed us to examine whether perceived economic pressure and asset-based living standards showed independent associations with child trafficking indicators.

#### Household-level covariates.

Household-level covariates were selected to reflect household composition, child vulnerability, and geographic context. Household size was defined as the total number of members listed on the household roster and was standardized for regression analyses. In the primary household-level model, household size was included as a continuous standardized covariate.

We also constructed two household-level child vulnerability indicators from rostered children aged 5–17. The first indicator identified households in which at least one rostered child aged 5–17 was not currently enrolled in formal school. The second indicator identified households in which at least one rostered child aged 5–17 had lost one or both parents. These variables were included because school non-enrollment and orphanhood represent salient child vulnerability factors in this context.

District fixed effects were included in all multivariable models, with Kenema as the reference district. These fixed effects adjust for stable cross-district differences in local labor markets, services, infrastructure, and social context that may be associated with both household conditions and child trafficking indicators.

As an alternative specification, we decomposed household size into the number of rostered children aged 5–17 and the number of non-child household members. This alternative household composition model was used to evaluate whether findings were sensitive to the way household size and child composition were represented.

#### Child-level covariates.

For child-level analyses, the outcome was the binary child trafficking indicator for each rostered child aged 5–17. Child-level covariates included age, sex, formal school enrollment, and orphan status. Child age was standardized. Sex was coded as female versus male. Formal school enrollment was coded 1 if the child was currently enrolled in formal school and 0 otherwise. Orphan status was coded 1 if the child had lost one or both parents and 0 otherwise.

Koranic school or madrassa enrollment was included in a sensitivity model rather than in the primary child-level model. This was done because Koranic or madrassa enrollment may capture a distinct educational and caregiving context that is substantively important but not equivalent to formal school enrollment.

Child-level models also included household-level economic strain, household wealth, household size, and district fixed effects. This specification allowed us to assess whether household-level economic conditions were associated with child-level trafficking indicators after accounting for child age, sex, schooling, orphanhood, and district context. Additional sensitivity models replaced overall household size with the number of children aged 5–17 and the number of non-child household members.

### Analytic strategy

#### Descriptive analysis.

Descriptive statistics were computed for two linked analytic samples. The household-level analytic sample included households with at least one rostered child aged 5–17 and a defined household-level trafficking indicator. The child-level analytic sample included rostered children aged 5–17 in those households. We summarized household characteristics overall and by household trafficking status, and child characteristics overall and by child trafficking status. Household-level child trafficking was defined as the presence of at least one child aged 5–17 with any trafficking indicator in the household. Child-level trafficking was defined as a binary indicator for whether the child had any trafficking indicator.

Descriptive statistics included household size, number of rostered children aged 5–17, number and proportion of children with trafficking indicators, subjective economic strain, objective material wealth, school enrollment vulnerability, orphan presence, child age, child sex, formal school enrollment, Koranic school or madrassa enrollment, orphan status, and district. Continuous variables are reported as means and standard deviations. Binary and categorical variables are reported as frequencies and percentages. Descriptive estimates were unweighted. The regression analyses also report unweighted associations in the eligible analytic household and child samples. Because the analyses focused on associations among households and rostered children with defined trafficking indicators rather than producing district-population prevalence estimates, the primary adjustment for the multistage design focused on dependence within enumeration areas. Clustering was addressed through enumeration-area-clustered standard errors in the primary models and random-intercept models in sensitivity analyses.

#### Model specifications.

For the primary household-level model, we estimated:


$$\begin{array}{cc} ogit\left\{P(Y_{h}=\text{1})\right\} & =\beta_{\text{0}}+\beta_{\text{1}}{Strain}_{h}+\beta_{\text{2}}{Wealth}_{h}+\beta_{\text{3}}{HouseholdSize}_{h}\\ & \;+\beta_{\text{4}}{OutOfSchool}_{h}+\beta_{\text{5}}{OrphanHousehold}_{h}+\delta^{\prime }{District}_{h} \end{array}$$ (1)

where $Y_{h}$ denotes whether household h had at least one rostered child aged 5–17 with one or more trafficking indicators. ${\text{Strain}}_{h}$, ${\text{Wealth}}_{h}$, and ${\text{HouseholdSize}}_{h}$ are standardized household-level measures. ${\text{OutOfSchool}}_{h}$ indicates any rostered child aged 5–17 in household h was not enrolled in formal school. ${\text{OrphanHousehold}}_{h}$ indicates whether any rostered child aged 5–17 in household h had lost one or both parents. ${\text{District}}_{h}$ is a vector of district indicators, with Kenema as the reference district.

For the grouped-binomial household model, we specified the outcome as:


$$\begin{array}{c} C_{h}\sim \text{ Binomial }(N_{h},\;p_{h}) \end{array}$$

and estimated:


$$\begin{array}{c} ogit\left(p_{h}\right)=\beta_{\text{0}}+\beta_{\text{1}}{Strain}_{h}+\beta_{\text{2}}{Wealth}_{h}+\beta_{\text{3}}{HouseholdSize}_{h}+\beta_{\text{4}}{OutOfSchool}_{h}+\beta_{\text{5}}{OrphanHousehold}_{h}+\delta^{\prime }{District}_{h} \end{array}$$ (2)

where $C_{h}$ is the number of rostered children aged 5–17 with trafficking indicators in household *h*, and $N_{h}$ is the total number of rostered children aged 5–17 in that household.

For the primary child-level model, we estimated:


$$\begin{array}{c} ogit\left\{P(Y_{ih}=\text{1})\right\}=\gamma_{\text{0}}+\gamma_{\text{1}}{Strain}_{h}+\gamma_{\text{2}}{Wealth}_{h}+\gamma_{\text{3}}{HouseholdSize}_{h}+\gamma_{\text{4}}{Age}_{ih}\\ +\gamma_{\text{5}}{Female}_{ih}+\;\gamma_{\text{6}}{FormalSchool}_{ih}+\gamma_{\text{7}}{OrphanChild}_{ih}+\eta \text{‘}{District}_{h} \end{array}$$ (3)

where $Y_{ih}$ indicates whether child i in household h had one or more trafficking indicators. ${\text{Age}}_{ih}$ is standardized child age, ${\text{Female}}_{ih}$ indicates female sex, ${\text{FormalSchool}}_{ih}$ indicates current formal school enrollment, and ${\text{OrphanChild}}_{ih}$ indicates whether the child had lost one or both parents. Standard errors for the primary models were clustered at the enumeration-area level.

#### Primary household-level model.

The primary household-level analysis used logistic regression to model the binary household trafficking outcome. The outcome was coded 1 if at least one rostered child aged 5–17 in the household had one or more trafficking indicators and 0 otherwise. Predictors included standardized subjective economic strain, standardized objective material wealth, standardized household size, an indicator for any child aged 5–17 not enrolled in formal school, an indicator for any orphan child aged 5–17 in the household, and district fixed effects. Kenema was used as the reference district.

Because the survey used a multistage cluster sampling design, standard errors in the primary regression models were clustered at the enumeration-area level. Odds ratios, 95% confidence intervals, and p-values are reported. Continuous predictors were standardized before model estimation, so odds ratios for these predictors represent the association with a one-standard-deviation difference in the predictor.

#### Alternative household-level outcome model.

To evaluate whether findings were sensitive to the aggregation of child-level indicators into a binary household outcome, we estimated a grouped-binomial household model. This model treated the number of children with trafficking indicators in each household as the numerator and the number of rostered children aged 5–17 in the household as the denominator. The grouped-binomial model therefore estimated associations with the proportion of children in the household who had trafficking indicators, rather than only whether the household had at least one affected child.

The grouped-binomial model included the same household-level predictors as the primary household-level model: standardized subjective economic strain, standardized objective material wealth, standardized household size, any child not enrolled in formal school, any orphan child in the household, and district fixed effects. Standard errors were clustered at the enumeration-area level.

#### Child-level model.

To further address the possibility that household-level aggregation obscured child-level heterogeneity, we estimated child-level logistic regression models using the binary child trafficking indicator as the outcome. The primary child-level model included household-level economic strain, household-level material wealth, household size, child age, child sex, formal school enrollment, orphan status, and district fixed effects. Household-level continuous predictors and child age were standardized. Kenema was again used as the reference district.

Because children were nested within households and households were nested within enumeration areas, the primary child-level model used standard errors clustered at the enumeration-area level. This approach aligned the child-level analysis with the primary sampling unit used in the household-level models.

#### Sensitivity analyses.

We conducted several sensitivity analyses to evaluate the robustness of the findings. First, we estimated alternative household composition models in which overall household size was replaced by two separate variables: the number of rostered children aged 5–17 and the number of non-child household members. This specification was used to assess whether the results depended on representing household composition as total household size.

Second, we estimated a child-level sensitivity model that added Koranic school or madrassa enrollment. This variable was not included in the primary child-level model because it reflects a distinct educational and caregiving context rather than formal school enrollment.

Third, we estimated random-intercept sensitivity models to assess the influence of clustering. Household-level sensitivity models included enumeration-area random intercepts. Child-level sensitivity models included both enumeration-area and household random intercepts. These models were used to evaluate whether substantive conclusions were consistent when clustering was modeled directly rather than addressed through enumeration-area-clustered standard errors.

#### Missing data.

The analyses used complete-case estimation because missingness in the final analytic variables was minimal. The primary household-level model included 2,428 of 2,429 eligible households, and the primary child-level model included 5,997 of 5,999 eligible children. Missingness and complete-case counts are reported in S3 and S4 Tables.

## Results

### Descriptive characteristics of the household and child analytic samples

Table 1 presents descriptive characteristics for the household-level and child-level analytic samples. The household-level analytic sample included 2,429 households with at least one rostered child aged 5–17. Among these households, 1,194 households, or 49.2%, had at least one child with one or more trafficking indicators. The mean household size was 5.93 members (SD = 2.27), and households included an average of 2.47 rostered children aged 5–17 (SD = 1.46). On average, households had 0.83 children with trafficking indicators (SD = 1.11), corresponding to a mean within-household proportion of 0.37 (SD = 0.43).

**Table 1:** Descriptive characteristics of the household and child analytic samples.

Panel A. Household-level analytic sample			
Characteristic	Overall	No household CT	Household CT
Households, n	2,429	1,235	1,194
Household with at least one CT-positive child, n (%)	1,194 (49.2%)	0 (0.0%)	1,194 (100.0%)
Household size, mean (SD)	5.93 (2.27)	5.86 (2.30)	6.02 (2.24)
Children aged 5–17, mean (SD)	2.47 (1.46)	2.40 (1.45)	2.55 (1.47)
CT-positive children, mean (SD)	0.83 (1.11)	0.00 (0.00)	1.70 (1.02)
Proportion of CT-positive children, mean (SD)	0.37 (0.43)	0.00 (0.00)	0.76 (0.29)
Economic strain scale, mean (SD)	2.28 (0.74)	2.31 (0.75)	2.26 (0.72)
Material wealth z-score, mean (SD)	0.00 (1.00)	−0.10 (1.08)	0.10 (0.90)
Any child not enrolled in formal school, n (%)	541 (22.3%)	229 (18.5%)	312 (26.1%)
Any orphan child, n (%)	551 (22.7%)	226 (18.3%)	325 (27.2%)
Kailahun, n (%)	700 (28.8%)	358 (29.0%)	342 (28.6%)
Kenema, n (%)	1,010 (41.6%)	558 (45.2%)	452 (37.9%)
Kono, n (%)	719 (29.6%)	319 (25.8%)	400 (33.5%)
**Panel B. Child-level analytic sample**			
**Characteristic**	**Overall**	**Child CT negative**	**Child CT positive**
Children aged 5–17, n	5,999	3,974	2,025
CT-positive child, *n* (%)	2,025 (33.8%)	0 (0.0%)	2,025 (100.0%)
Age, mean (SD)	10.67 (3.72)	9.96 (3.66)	12.08 (3.42)
Female, *n* (%)	3,112 (51.9%)	2,136 (53.7%)	976 (48.2%)
Enrolled in formal school, *n* (%)	5,290 (88.2%)	3,596 (90.5%)	1,694 (83.7%)
Enrolled in Koranic school or madrassa, *n* (%)	1,321 (22.0%)	838 (21.1%)	483 (23.9%)
Orphan child, *n* (%)	941 (15.7%)	540 (13.6%)	401 (19.8%)
Kailahun, *n* (%)	1,740 (29.0%)	1,169 (29.4%)	571 (28.2%)
Kenema, *n* (%)	2,731 (45.5%)	1,973 (49.6%)	758 (37.4%)
Kono, *n* (%)	1,528 (25.5%)	832 (20.9%)	696 (34.4%)

*Note* CT = child trafficking indicators. Panel A describes the household-level analytic sample, defined as households with at least one rostered child aged 5–17. Household CT indicates that at least one rostered child aged 5–17 in the household had one or more trafficking indicators according to the operational definition. Panel B describes the child-level analytic sample of rostered children aged 5–17. Child CT indicates that the child had one or more trafficking indicators. Economic strain is the six-item household economic strain scale, with higher scores indicating greater perceived economic strain. Material wealth is a standardized household-level asset-based wealth score, with higher scores indicating greater material wealth. Formal school enrollment refers to current enrollment in formal schooling. Percentages are unweighted.

Households with at least one child with trafficking indicators differed from households without such indicators on several descriptive characteristics. They had slightly larger household size (M = 6.02 vs. 5.86) and slightly more rostered children aged 5–17 (M = 2.55 vs. 2.40). They also had a higher mean material wealth z-score (0.10 vs. −0.10). Child vulnerability indicators were more common among households with trafficking indicators: 26.1% had at least one child not enrolled in formal school, compared with 18.5% of households without trafficking indicators; 27.2% had at least one orphan child, compared with 18.3% of households without trafficking indicators. District composition also differed. Kono represented 33.5% of households with trafficking indicators but 25.8% of households without trafficking indicators.

The child-level analytic sample included 5,999 rostered children aged 5–17. Of these children, 2,025, or 33.8%, had one or more trafficking indicators. Children with trafficking indicators were older on average than children without trafficking indicators (M = 12.08 vs. 9.96 years). They were less likely to be female (48.2% vs. 53.7%), less likely to be enrolled in formal school (83.7% vs. 90.5%), and more likely to be orphan children (19.8% vs. 13.6%). Kono accounted for 34.4% of children with trafficking indicators but 20.9% of children without trafficking indicators.

### Primary household-level binary model

Table 2 presents the primary household-level logistic regression model estimating associations with whether a household had at least one rostered child aged 5–17 with one or more trafficking indicators. Standard errors were clustered at the enumeration-area level, and all models included district fixed effects with Kenema as the reference district.

**Table 2:** Household-level logistic regression modeling any child trafficking indicator.

Predictor	Odds ratio	95% CI	*p*-value
Economic strain, standardized	0.93	[0.76, 1.14]	.487
Material wealth, standardized	1.21	[1.02, 1.44]	.027
Household size, standardized	1.15	[0.99, 1.34]	.067
Any child not enrolled in formal school	1.35	[1.02, 1.78]	.035
Any orphan child in household	1.66	[1.30, 2.11]	<.001
Kailahun vs. Kenema	1.15	[0.60, 2.19]	.681
Kono vs. Kenema	1.73	[0.92, 3.24]	.087

*Note* The outcome is household-level child trafficking indicator status, coded 1 if at least one rostered child aged 5–17 in the household had one or more trafficking indicators and 0 otherwise. The model was estimated using logistic regression with standard errors clustered at the enumeration-area level. Kenema was the reference district. Continuous predictors were standardized, so odds ratios for economic strain, material wealth, and household size represent the association with a one-standard-deviation difference in the predictor. Economic strain is the six-item household economic strain scale, with higher values indicating greater perceived economic strain. Material wealth is the standardized asset-based wealth score, with higher values indicating greater material wealth. CI = confidence interval.

Subjective economic strain was not significantly associated with the household-level trafficking indicator in the primary model (OR = 0.93, 95% CI [0.76, 1.14], *p* = .487). In contrast, objective material wealth was positively associated with the household-level trafficking indicator (OR = 1.21, 95% CI [1.02, 1.44], *p* = .027), indicating higher odds of household trafficking indicators for households with higher asset-based material wealth, net of subjective economic strain, household size, child vulnerability indicators, and district.

Household size was positively associated with the outcome but did not reach conventional statistical significance in the primary model (OR = 1.15, 95% CI [0.99, 1.34], *p* = .067). Household-level child vulnerability indicators were more consistently associated with the outcome. Households with at least one child not enrolled in formal school had higher odds of having a child with trafficking indicators (OR = 1.35, 95% CI [1.02, 1.78], *p* = .035). Households with at least one orphan child also had higher odds of having a child with trafficking indicators (OR = 1.66, 95% CI [1.30, 2.11], *p* < .001).

After adjustment for household economic conditions, household size, and child vulnerability indicators, Kailahun did not differ significantly from Kenema (OR = 1.15, 95% CI [0.60, 2.19], *p* = .681). Kono showed higher odds than Kenema, although this contrast was marginal in the household-level binary model (OR = 1.73, 95% CI [0.92, 3.24], *p* = .087).

### Aggregation robustness analyses

Table 3 presents two additional models used to evaluate whether the findings were sensitive to household-level aggregation of child trafficking indicators. The first was a grouped-binomial household model modeling the number of children with trafficking indicators out of the number of rostered children aged 5–17 in each household. The second was a child-level logistic regression model predicting each child’s binary trafficking indicator.

**Table 3:** Aggregation robustness models for child trafficking indicators.

Predictor	Grouped-binomial household model OR [95% CI]	*p*-value	Child-level logistic model OR [95% CI]	*p*-value
Economic strain, standardized	0.92 [0.75, 1.13]	.442	0.89 [0.71, 1.11]	.296
Material wealth, standardized	1.21 [1.00, 1.46]	.049	1.29 [1.06, 1.58]	.013
Household size, standardized	0.89 [0.77, 1.02]	.087	0.88 [0.76, 1.02]	.099
Any child not enrolled in formal school	1.35 [1.04, 1.76]	.026	Not included	Not included
Any orphan child in household	1.39 [1.11, 1.73]	.003	Not included	Not included
Child age, standardized	Not included	Not included	1.87 [1.67, 2.10]	<.001
Female child	Not included	Not included	0.81 [0.71, 0.92]	.001
Child enrolled in formal school	Not included	Not included	0.68 [0.52, 0.91]	.008
Orphan child	Not included	Not included	1.34 [1.08, 1.67]	.009
Kailahun vs. Kenema	1.10 [0.54, 2.24]	.787	1.08 [0.50, 2.34]	.841
Kono vs. Kenema	2.11 [1.14, 3.91]	.018	2.07 [1.05, 4.10]	.037

*Note* The grouped-binomial household model estimates associations with the number of children with trafficking indicators out of the total number of rostered children aged 5–17 in each household. The child-level logistic model predicts whether each rostered child aged 5–17 had one or more trafficking indicators. Both models included district fixed effects, with Kenema as the reference district, and used standard errors clustered at the enumeration-area level. Continuous predictors were standardized. Economic strain is the six-item household economic strain scale, with higher values indicating greater perceived economic strain. Material wealth is the standardized asset-based wealth score, with higher values indicating greater material wealth. “Not included” indicates that the predictor was not defined at that level of analysis or was replaced by the corresponding child-level or household-level measure. OR = odds ratio; CI = confidence interval.

In the grouped-binomial household model, subjective economic strain was again not significantly associated with the proportion of children in the household who had trafficking indicators (OR = 0.92, 95% CI [0.75, 1.13], *p* = .442). Objective material wealth remained positively associated with the outcome (OR = 1.21, 95% CI [1.00, 1.46], *p* = .049). Households with at least one child not enrolled in formal school had higher odds of child trafficking indicators in the grouped-binomial model (OR = 1.35, 95% CI [1.04, 1.76], *p* = .026), as did households with at least one orphan child (OR = 1.39, 95% CI [1.11, 1.73], *p* = .003). Kono was significantly higher than Kenema in this model (OR = 2.11, 95% CI [1.14, 3.91], *p* = .018), whereas Kailahun did not differ significantly from Kenema (OR = 1.10, 95% CI [0.54, 2.24], *p* = .787).

The child-level model showed a similar pattern for household-level economic variables and district context, while also identifying child-level correlates. Subjective economic strain was not significantly associated with child-level trafficking indicators (OR = 0.89, 95% CI [0.71, 1.11], *p* = .296). Objective material wealth was positively associated with child-level trafficking indicators (OR = 1.29, 95% CI [1.06, 1.58], *p* = .013). Older children had substantially higher odds of trafficking indicators (OR = 1.87, 95% CI [1.67, 2.10], *p* < .001). Female children had lower odds than male children (OR = 0.81, 95% CI [0.71, 0.92], *p* = .001). Children enrolled in formal school had lower odds of trafficking indicators (OR = 0.68, 95% CI [0.52, 0.91], *p* = .008), whereas orphan children had higher odds (OR = 1.34, 95% CI [1.08, 1.67], *p* = .009). Kono remained significantly higher than Kenema in the child-level model (OR = 2.07, 95% CI [1.05, 4.10], *p* = .037), whereas Kailahun did not differ from Kenema (OR = 1.08, 95% CI [0.50, 2.34], *p* = .841).

Together, the grouped-binomial and child-level models indicate that the main findings were not solely an artifact of coding households as positive when any child had trafficking indicators. Across outcome specifications, subjective economic strain was not a robust positive correlate, whereas objective material wealth, school enrollment vulnerability, orphanhood, child age, and Kono district were more consistent correlates of trafficking indicators.

### Sensitivity analyses

Several sensitivity analyses were conducted to assess the robustness of the findings. First, alternative household composition models replaced overall household size with two separate variables: the number of rostered children aged 5–17 and the number of non-child household members. The overall pattern of findings was similar. In the child-level alternative household composition model, the number of children aged 5–17 was negatively associated with child-level trafficking indicators, whereas the number of non-child household members was not statistically significant. This pattern is consistent with the distinction between household-level any-child outcomes and child-level risk. Households with more children may have more opportunities to contain at least one child with trafficking indicators, but the estimated odds for an individual child can differ after child age, schooling, orphanhood, and district are taken into account.

Second, adding Koranic school or madrassa enrollment to the child-level model did not change the main pattern of results. Koranic school or madrassa enrollment was positively associated with child-level trafficking indicators in the sensitivity model (OR = 1.43, 95% CI [1.01, 2.02], *p* = .045), while objective material wealth, child age, sex, formal school enrollment, orphanhood, and Kono district remained associated with the outcome.

Third, random-intercept models were estimated to evaluate clustering. Household-level random-intercept models included enumeration-area random intercepts. Child-level random-intercept models included both enumeration-area and household random intercepts. These models showed substantial clustering, especially in the child-level analyses. In the primary child-level random-intercept model, the total cluster intraclass correlation was approximately 0.70, with variation attributable to both enumeration areas and households. Results from the random-intercept models generally supported the importance of clustering and the consistent associations of material wealth, child age, sex, formal school enrollment, and orphanhood. However, subjective economic strain was more sensitive to modeling approach. It was not statistically significant in the primary enumeration-area-clustered household and child models, but became negative and statistically significant in the conditional random-intercept child-level models. We therefore interpret subjective economic strain cautiously and do not characterize it as a robust positive correlate of trafficking indicators. Alternative EA-clustered specifications, random-intercept models, and ICC summaries are reported in S5–S7 Tables.

The analyses provide converging evidence across household-level, grouped-binomial, and child-level outcome specifications. The primary household-level binary model is useful for identifying households in which at least one child had trafficking indicators. The grouped-binomial and child-level models address the aggregation of child-level indicators into household-level outcomes and show that several findings were consistent across levels of analysis. Overall, objective material wealth, school enrollment vulnerability, orphanhood, child age, and Kono district were more consistent correlates of trafficking indicators than subjective economic strain. Subjective economic strain showed good scale reliability but was not a stable positive correlate in the primary enumeration-area-clustered models.

## Discussion

This study examined household- and child-level economic and social correlates of child trafficking indicators in Sierra Leone’s Eastern Province. The analyses used three complementary outcome specifications: a household-level binary indicator, a grouped-binomial household outcome based on the number of children with trafficking indicators out of all rostered children aged 5–17, and a child-level trafficking indicator. This approach allowed us to retain the policy relevance of identifying households in which at least one child had trafficking indicators while also addressing the possibility that a household-level binary outcome could obscure variation across children within households.

Across these models, several findings were consistent. Objective material wealth, school enrollment vulnerability, orphanhood, child age, and district context showed more consistent associations with trafficking indicators than subjective economic strain. Subjective economic strain showed good scale reliability, but it was not a robust positive correlate in the primary enumeration-area-clustered household and child models. In contrast, material wealth was positively associated with trafficking indicators across household-level, grouped-binomial, and child-level models. Households with at least one child not enrolled in formal school and households with at least one orphan child had higher odds of household trafficking indicators. At the child level, older children and orphan children had higher odds of trafficking indicators, whereas female children and children enrolled in formal school had lower odds. Kono also showed elevated trafficking indicators relative to Kenema, especially in the grouped-binomial and child-level models.

### Economic conditions and child trafficking indicators

The economic findings require careful interpretation. The original expectation was that greater subjective economic strain and lower material wealth would be associated with higher trafficking risk. However, the models did not support a simple poverty-gradient interpretation. Subjective economic strain was not statistically significant in the primary household-level, grouped-binomial, or child-level enumeration-area-clustered models. It became negative and statistically significant in some conditional random-intercept sensitivity models, indicating that its estimated association was sensitive to how clustering was modeled. For this reason, subjective economic strain should not be interpreted as a stable positive predictor of trafficking indicators in these data.

The positive association between objective material wealth and trafficking indicators was also unexpected and should not be interpreted as evidence that wealth causes trafficking. One possible explanation is that the operational definition captured multiple forms of hazardous and exploitative work, including work in sectors such as mining, fishing, portering, manufacturing, and construction. In this context, somewhat more materially resourced households may have greater access to labor opportunities, productive assets, mobility, or local economic networks in which children’s work is more likely to occur or be reported. Another possibility is that the wealth score captures participation in more monetized or labor-intensive local economies rather than protection from child labor and trafficking indicators. The persistence of Kono’s elevated risk further suggests that district-level economic context may shape the relationship between household resources and child work-related trafficking indicators. These explanations remain hypotheses because the survey was cross-sectional and did not directly measure labor demand, recruitment networks, household income flows, or the specific economic activities linking assets to child work.

The distinction between subjective strain and objective wealth is still substantively useful. Subjective strain captures perceived difficulty meeting household needs, whereas asset-based wealth captures observed material living standards. Their different associations with trafficking indicators suggest that these measures should not be treated as interchangeable proxies for poverty. The results point to the need for more nuanced economic measurement in trafficking research, especially in settings where household assets may index both material advantage and integration into local labor markets.

### Schooling, orphanhood, and child-level vulnerability

School enrollment and orphanhood were more consistent vulnerability markers than subjective economic strain. At the household level, having at least one child not enrolled in formal school was associated with higher odds of having at least one child with trafficking indicators. At the child level, formal school enrollment was associated with lower odds of trafficking indicators. These findings are consistent with the idea that school enrollment may be protective, may reduce children’s availability for hazardous work, or may reflect broader household and community conditions that support child protection. Because the data are cross-sectional, however, the direction of association cannot be determined. Trafficking-related work may disrupt schooling, school non-enrollment may increase exposure to hazardous work, or both may be driven by underlying household and community vulnerability.

Orphanhood also emerged as a consistent correlate. Households with at least one orphan child had higher odds of household trafficking indicators, and orphan children had higher odds of child-level trafficking indicators. This finding highlights the importance of caregiving structure and child protection context. Children who have lost one or both parents may face reduced supervision, greater economic pressure to contribute to household survival, placement in extended households, or increased vulnerability to exploitative work arrangements. These mechanisms were not directly tested, but the consistency of the orphanhood association across household and child models suggests that orphan status should be considered in prevention, screening, and referral systems. Importantly, orphanhood should not be interpreted as an exogenous household characteristic. It may instead reflect caregiving disruption, informal kinship-care arrangements, or household embeddedness in the broader system of child movement and placement that can also generate trafficking vulnerability.

Child age and sex were also important in the child-level models. Older children had substantially higher odds of trafficking indicators, which is consistent with greater involvement in work as children approach adolescence. Female children had lower odds than male children in these models. This pattern should be interpreted in relation to the operational definition, which included multiple labor-sector and hazardous-work indicators. It may reflect gendered differences in the types of work captured by the survey instrument, differential visibility of boys’ and girls’ labor, or under-detection of less visible forms of exploitation affecting girls.

### District context and clustering

District context was an important part of the findings. Kono showed higher trafficking indicators than Kenema in the grouped-binomial and child-level models, and it was descriptively the district with the highest household- and child-level prevalence. This pattern suggests that household characteristics alone do not fully explain geographic differences in trafficking indicators. Kono’s local economic structure, including mining-related labor markets, mobility patterns, and informal work opportunities, may contribute to elevated risk. However, these mechanisms were not measured directly and should be treated as contextual hypotheses rather than confirmed pathways.

The random-intercept sensitivity analyses also showed substantial clustering, particularly in the child-level models. The primary child-level random-intercept model indicated that a large share of unexplained variation was attributable to enumeration areas and households. This finding supports the decision to account for the multistage sample design and suggests that trafficking indicators are not randomly distributed across children. Instead, they cluster within places and households. Future research should examine community-level and household-level mechanisms more directly, including local labor demand, school access, social protection coverage, caregiver structure, and child protection referral capacity.

### Implications for policy and practice

The findings support a multi-level prevention approach. Because school non-enrollment and orphanhood were consistent correlates, prevention programs should prioritize school access, school retention, and support for children in disrupted caregiving arrangements. School-fee support, re-enrollment assistance, case management for out-of-school children, and monitoring of orphaned or separated children may be especially relevant. These strategies should be linked to child protection referral systems rather than implemented as isolated education interventions.

The positive association between material wealth and trafficking indicators suggests that targeting based only on asset poverty may miss some households and children exposed to trafficking-related work. Prevention systems should therefore combine economic vulnerability criteria with child-level and context-specific indicators, such as school non-enrollment, orphanhood, age, work sector, hazardous work conditions, and district-level risk. In settings where trafficking indicators are tied to local labor markets, programs may need to monitor households and communities connected to high-risk economic sectors, not only the poorest households.

District-level differences also indicate the need for place-based strategies. In higher-prevalence districts such as Kono, household supports should be complemented by community-level and labor-market interventions. These may include child protection outreach in high-risk work settings, school retention initiatives, safe reporting pathways, labor oversight in informal sectors, and referral coordination among schools, community leaders, social welfare actors, and local authorities. Because the present study did not directly measure district-level mechanisms, such strategies should be piloted and evaluated rather than assumed to be effective.

### Strengths and limitations

This study has several strengths. First, it used a large household survey covering 2,429 households and 5,999 rostered children aged 5–17 in three districts of Sierra Leone’s Eastern Province. Second, the analysis reconstructed child-level trafficking indicators from the operational definition and examined outcomes at multiple levels. Third, the use of household binary, grouped-binomial, and child-level models directly addressed the concern that a household-level any-child indicator may obscure within-household heterogeneity. Fourth, the analyses accounted for the multistage design using enumeration-area-clustered standard errors and random-intercept sensitivity models.

Several limitations should also be noted. Because the analysis was based on rostered children reported by adult household respondents, children who were absent from the household, not reported as household members, or already living elsewhere in exploitative arrangements may not have been fully captured. This could lead to underestimation of child trafficking indicators, particularly for children who had left the household or were not recognized by respondents as still connected to the household. In addition, the study relied on adult caregiver reports rather than direct child interviews. Underreporting or misclassification may have occurred, especially for hidden or stigmatized forms of exploitation. The trafficking indicator was based on a survey operational definition and should not be interpreted as a legal determination of trafficking victimization. The measure combined hazardous sectors, hazardous work conditions, and force, fraud, coercion, and restriction indicators. These components may reflect different mechanisms and may have different correlates. Future research should examine these subdomains separately when sample size permits.

The cross-sectional design limits causal interpretation. The observed associations cannot determine whether household conditions lead to trafficking indicators, whether children’s work experiences alter household conditions, or whether both are shaped by unmeasured community and labor-market factors. The positive association between material wealth and trafficking indicators, in particular, requires further study using longitudinal data, more detailed measures of income and assets, and direct measures of local labor demand and child work sectors.

The study also did not include all potentially relevant variables. Some measures that would be useful for mechanism testing, such as detailed caregiver employment, household income, adult labor capacity, social protection participation, recruitment pathways, and community-level child protection resources, were not available in the analytic files used for the models. In addition, although random-intercept models demonstrated strong clustering, the present study could not fully explain why certain enumeration areas or districts had higher trafficking indicators. Because the models were estimated without sampling weights, the findings should be interpreted as unweighted associations in the analytic household and child samples rather than weighted district-level population estimates.

## Conclusion

In summary, the current study shows that child trafficking indicators in Sierra Leone’s Eastern Province are patterned by household, child, and district context. The household-level binary outcome remains useful for identifying households in which at least one child has trafficking indicators, but grouped-binomial and child-level models show that conclusions are not solely an artifact of household-level aggregation. Across models, objective material wealth, school enrollment vulnerability, orphanhood, child age, and Kono district were more consistent correlates than subjective economic strain. These findings suggest that prevention strategies should move beyond a simple poverty-only framework and integrate household support, school access, child protection for orphaned or out-of-school children, and place-based responses in higher-risk districts.

## Supporting information

S1 Table Model sample summary. Summary of the analytic level, model sample, number of children, households, enumeration areas, and districts, and the prevalence of household-level and child-level trafficking indicators across the primary and sensitivity analyses. CT = child trafficking indicators.
(DOCX)

S2 Table Economic strain and wealth diagnostics. Description of the economic strain items, response coding, scale construction, reliability estimates, descriptive statistics, wealth-score orientation, and correlations among economic strain, material wealth, and household-level child trafficking indicators. CT = child trafficking indicators.
(DOCX)

S3 Table Missingness of key analytic variables. Numbers and percentages of missing observations for the household-level and child-level variables used in the revised analyses. CT = child trafficking indicators.
(DOCX)

S4 Table Complete-case sample sizes for primary and sensitivity models. Total analytic sample sizes, complete-case sample sizes, and complete-case percentages for the primary household-level model, the alternative household-composition model, and the primary child-level model.
(DOCX)

S5 Table Alternative EA-clustered model specifications. Odds ratios, 95% confidence intervals, and p values from alternative household-composition models and child-level sensitivity models. All models used standard errors clustered at the enumeration-area level and included district fixed effects, with Kenema as the reference district. EA = enumeration area; OR = odds ratio; CI = confidence interval.
(DOCX)

S6 Table Random-intercept sensitivity models. Odds ratios, 95% confidence intervals, and p values from household-level models with enumeration-area random intercepts and the child-level model with enumeration-area and household random intercepts. All models included district fixed effects, with Kenema as the reference district. EA = enumeration area; OR = odds ratio; CI = confidence interval.
(DOCX)

S7 Table Random-intercept intraclass correlation summary. Summary of the random effects, enumeration-area intraclass correlations, household intraclass correlations, total cluster intraclass correlations, and convergence status for the random-intercept sensitivity models. EA = enumeration area; ICC = intraclass correlation coefficient.
(DOCX)
