# Supplementary material for: Household and child correlates of child trafficking indicators in Eastern Sierra Leone
Source: PLoS One. 2026 Oct 5;21(10):e0347898. doi: 10.1371/journal.pone.0347898 (PMC13637909; doi:10.1371/journal.pone.0347898)
Supplement: Supplementary file 1 [file pone.0347898.s001.docx]

**Household and Child Correlates of Child Trafficking Indicators in Eastern Sierra Leone**

**S1 Table. Model sample summary**

| Analytic level | Model sample | N children | *N* households | *N* enumeration areas | *N* districts | Household CT prevalence | Child CT prevalence |
| --- | --- | --- | --- | --- | --- | --- | --- |
| Household | Household binary primary model | Not applicable | 2,428 | 113 | 3 | 49.2% | 33.8% |
| Household | Household binary alternative composition model | Not applicable | 2,427 | 113 | 3 | 49.2% | 33.7% |
| Household | Grouped-binomial household primary model | Not applicable | 2,428 | 113 | 3 | 49.2% | 33.8% |
| Household | Grouped-binomial household alternative composition model | Not applicable | 2,427 | 113 | 3 | 49.2% | 33.7% |
| Child | Child-level primary model | 5,997 | 2,428 | 113 | 3 | Not applicable | 33.8% |
| Child | Child-level Koranic school sensitivity model | 5,997 | 2,428 | 113 | 3 | Not applicable | 33.8% |
| Child | Child-level alternative household composition model | 5,992 | 2,427 | 113 | 3 | Not applicable | 33.7% |
| Child | Child-level no-formal-schooling sensitivity model | 5,997 | 2,428 | 113 | 3 | Not applicable | 33.8% |

*Note.* CT = child trafficking indicators. Household CT prevalence refers to the proportion of households with at least one rostered child aged 5 to 17 with one or more trafficking indicators. Child CT prevalence refers to the proportion of rostered children aged 5 to 17 with one or more trafficking indicators. Household models used household-level outcomes; child-level models used child-level outcomes. The grouped-binomial household models used the number of children with trafficking indicators out of the number of rostered children aged 5 to 17 in each household. Alternative composition models replaced overall household size with separate standardized counts of children aged 5 to 17 and non-child household members. The Koranic school sensitivity model added Koranic school or madrassa enrollment to the primary child-level model. The no-formal-schooling sensitivity model omitted formal school enrollment from the child-level model.
