# Supplementary material for: Household and child correlates of child trafficking indicators in Eastern Sierra Leone
Source: PLoS One. 2026 Oct 5;21(10):e0347898. doi: 10.1371/journal.pone.0347898 (PMC13637909; doi:10.1371/journal.pone.0347898)
Supplement: Supplementary file 2 [file pone.0347898.s002.docx]

**Household and Child Correlates of Child Trafficking Indicators in Eastern Sierra Leone**

**S2 Table. Economic strain and wealth diagnostics**

| Diagnostic | Result |
| --- | --- |
| Selected economic strain items | D1: household is well off by local standards; D2: household finds it difficult to live on current income; D3: enough food; D4: enough money for school fees; D5: enough money for clothing; D6: enough money for medicine |
| Number of economic strain items | 6 |
| Economic strain item response scale | 1 = strongly agree to 5 = strongly disagree |
| Economic strain scoring direction | Higher scores indicate greater perceived economic strain |
| Economic strain coding | D2 was reverse-coded because stronger agreement with income difficulty indicates greater strain. D1 and D3–D6 were retained in their original direction because stronger disagreement indicates greater strain. |
| Missingness for D1–D6 raw items | 1 missing response per item in the household analytic sample |
| Economic strain scale score | Mean of the six keyed items; households with at least four observed items were assigned the mean of available keyed items |
| Complete cases for reliability analysis | 2,428 households |
| Six-item economic strain scale, raw alpha | 0.839 |
| Six-item economic strain scale, standardized alpha | 0.864 |
| Five-item sensitivity scale excluding D1, raw alpha | 0.838 |
| Five-item sensitivity scale excluding D1, standardized alpha | 0.870 |
| Mean six-item economic strain score, overall | 2.28 |
| SD six-item economic strain score, overall | 0.735 |
| Mean six-item economic strain score, households without CT indicators | 2.31 |
| Mean six-item economic strain score, households with CT indicators | 2.26 |
| Wealth score variable | Asset-based household wealth score |
| Wealth orientation check | Raw wealth score was negatively correlated with the family resource mean (r = -0.186) |
| Wealth orientation adjustment | Raw wealth score was multiplied by -1 so that higher values indicate greater material wealth |
| Missingness for oriented wealth score | 1 missing value in the household analytic sample |
| Correlation between economic strain and standardized wealth | *r* = -0.186 |
| Correlation between household CT indicator and economic strain | *r* = -0.033 |
| Correlation between household CT indicator and standardized wealth | *r* = 0.098 |
| Correlation between proportion of CT-positive children and economic strain | *r* = -0.008 |
| Correlation between proportion of CT-positive children and standardized wealth | *r* = 0.106 |

*Note.* CT = child trafficking indicators. Economic strain was scored so that higher values indicate greater perceived economic strain. The six-item scale included perceived local standing, income difficulty, food adequacy, school-fee adequacy, clothing adequacy, and medicine adequacy. The five-item scale excluding the local-standing item was examined as a sensitivity version and showed similar reliability. The wealth score was oriented so that higher values indicate greater objective material wealth. Correlations are descriptive and were computed using available cases in the household analytic sample.
