# Supplementary material for: Household and child correlates of child trafficking indicators in Eastern Sierra Leone
Source: PLoS One. 2026 Oct 5;21(10):e0347898. doi: 10.1371/journal.pone.0347898 (PMC13637909; doi:10.1371/journal.pone.0347898)
Supplement: Supplementary file 3 [file pone.0347898.s003.docx]

**Household and Child Correlates of Child Trafficking Indicators in Eastern Sierra Leone**

**S3 Table. Missingness of key analytic variables**

| Analytic level | Variable | N | Missing, n (%) | |
| --- | --- | --- | --- | --- |
| Household-level variables | Household CT indicator | 2,429 | 0 (0.0%) |  |
|  | Number of CT-positive children | 2,429 | 0 (0.0%) |  |
|  | Proportion of CT-positive children | 2,429 | 0 (0.0%) |  |
|  | Number of children aged 5 to 17 | 2,429 | 0 (0.0%) |  |
|  | Household size | 2,429 | 0 (0.0%) |  |
|  | Number of non-child household members | 2,429 | 1 (0.0%) |  |
|  | Economic strain scale, six-item mean | 2,429 | 1 (0.0%) |  |
|  | Economic strain, standardized | 2,429 | 1 (0.0%) |  |
|  | Economic strain scale, five-item sensitivity version | 2,429 | 1 (0.0%) |  |
|  | Material wealth score | 2,429 | 1 (0.0%) |  |
|  | Material wealth, standardized | 2,429 | 1 (0.0%) |  |
|  | District | 2,429 | 0 (0.0%) |  |
|  | Enumeration area | 2,429 | 0 (0.0%) |  |
|  | Any child not enrolled in formal school | 2,429 | 0 (0.0%) |  |
|  | Any orphan child in household | 2,429 | 0 (0.0%) |  |
| Child-level variables | Child CT indicator | 5,999 | 0 (0.0%) |  |
|  | Child age | 5,999 | 0 (0.0%) |  |
|  | Female child | 5,999 | 0 (0.0%) |  |
|  | Enrolled in formal school | 5,999 | 0 (0.0%) |  |
|  | Enrolled in Koranic school or madrassa | 5,999 | 0 (0.0%) |  |
|  | Orphan child | 5,999 | 0 (0.0%) |  |
|  | District | 5,999 | 0 (0.0%) |  |
|  | Enumeration area | 5,999 | 0 (0.0%) |  |
|  | Household identifier | 5,999 | 0 (0.0%) |  |

*Note.* CT = child trafficking indicators. Household-level variables were assessed among households with at least one rostered child aged 5 to 17. Child-level variables were assessed among rostered children aged 5 to 17 in those households. Percentages are rounded to one decimal place; values shown as 0.0% indicate fewer than 0.05% missing. Because missingness in the final analytic variables was minimal, the revised analyses used complete-case estimation rather than multiple imputation.
