# Supplementary material for: Household and child correlates of child trafficking indicators in Eastern Sierra Leone
Source: PLoS One. 2026 Oct 5;21(10):e0347898. doi: 10.1371/journal.pone.0347898 (PMC13637909; doi:10.1371/journal.pone.0347898)
Supplement: Supplementary file 4 [file pone.0347898.s004.docx]

**Household and Child Correlates of Child Trafficking Indicators in Eastern Sierra Leone**

**S4 Table. Complete-case sample sizes for primary and sensitivity models**

| Model dataset | Total analytic N | Complete-case N | Complete-case percentage |
| --- | --- | --- | --- |
| Household primary model | 2,429 households | 2,428 households | 100.0% |
| Household alternative composition model | 2,429 households | 2,427 households | 99.9% |
| Child primary model | 5,999 children | 5,997 children | 100.0% |

*Note.* The household primary model refers to the household-level logistic regression modeling whether a household had at least one rostered child aged 5 to 17 with one or more trafficking indicators. The household alternative composition model replaced standardized household size with separate standardized counts of children aged 5 to 17 and non-child household members. The child primary model refers to the child-level logistic regression modeling whether each rostered child aged 5 to 17 had one or more trafficking indicators. Complete-case percentages are rounded to one decimal place; 100.0% indicates that fewer than 0.05% of observations were excluded. Because complete-case loss was minimal, the revised analyses used complete-case estimation rather than multiple imputation.
