# Supplementary material for: Household and child correlates of child trafficking indicators in Eastern Sierra Leone
Source: PLoS One. 2026 Oct 5;21(10):e0347898. doi: 10.1371/journal.pone.0347898 (PMC13637909; doi:10.1371/journal.pone.0347898)
Supplement: Supplementary file 5 [file pone.0347898.s005.docx]

**Household and Child Correlates of Child Trafficking Indicators in Eastern Sierra Leone**

**S5 Table. Alternative EA-clustered model specifications**

| Predictor | Household binary, alternative composition OR [95% CI], p | Grouped-binomial household, alternative composition OR [95% CI], p | Child-level, Koranic school sensitivity OR [95% CI], p | Child-level, alternative household composition OR [95% CI], p |
| --- | --- | --- | --- | --- |
| Economic strain, standardized | 0.93 [0.76, 1.14], p = .492 | 0.89 [0.73, 1.08], p = .233 | 0.88 [0.71, 1.11],  p = .283 | 0.86 [0.69, 1.06], p = .154 |
| Material wealth, standardized | 1.21 [1.02, 1.44],  p = .027 | 1.20 [1.00, 1.46], p = .054 | 1.30 [1.06, 1.58],  p = .011 | 1.29 [1.05, 1.58], p = .013 |
| Household size, standardized | Not included | Not included | 0.87 [0.75, 1.02],  p = .079 | Not included |
| Number of children aged 5 to 17, standardized | 1.10 [0.96, 1.26],  p = .149 | 0.78 [0.68, 0.90], p < .001 | Not included | 0.80 [0.69, 0.94], p = .005 |
| Number of non-child household members, standardized | 1.10 [0.96, 1.26],  p = .157 | 1.09 [0.98, 1.22], p = .122 | Not included | 1.06 [0.95, 1.18], p = .323 |
| Any child not enrolled in formal school | 1.35 [1.02, 1.77],  p = .035 | 1.43 [1.09, 1.87], p = .010 | Not included | Not included |
| Any orphan child in household | 1.65 [1.29, 2.11],  p < .001 | 1.55 [1.25, 1.91], p < .001 | Not included | Not included |
| Child age, standardized | Not included | Not included | 1.88 [1.68, 2.11],  p < .001 | 1.87 [1.67, 2.09], p < .001 |
| Female child | Not included | Not included | 0.83 [0.73, 0.94],  p = .003 | 0.80 [0.70, 0.91], p < .001 |
| Child enrolled in formal school | Not included | Not included | 0.67 [0.51, 0.90],  p = .007 | 0.70 [0.53, 0.93], p = .013 |
| Child enrolled in Koranic school or madrassa | Not included | Not included | 1.43 [1.01, 2.02],  p = .045 | Not included |
| Orphan child | Not included | Not included | 1.34 [1.08, 1.67],  p = .008 | 1.43 [1.16, 1.75], p < .001 |
| Kailahun vs. Kenema | 1.14 [0.60, 2.17],  p = .683 | 1.18 [0.59, 2.34], p = .640 | 1.13 [0.52, 2.42],  p = .760 | 1.14 [0.54, 2.42], p = .726 |
| Kono vs. Kenema | 1.72 [0.92, 3.23],  p = .089 | 2.17 [1.16, 4.05], p = .015 | 2.15 [1.11, 4.19],  p = .024 | 2.12 [1.07, 4.21], p = .032 |

*Note.* OR = odds ratio; CI = confidence interval. All models used standard errors clustered at the enumeration-area level and included district fixed effects, with Kenema as the reference district. Continuous predictors were standardized. The household binary alternative composition model modeled whether a household had at least one rostered child aged 5 to 17 with one or more trafficking indicators, replacing overall household size with the number of children aged 5 to 17 and the number of non-child household members. The grouped-binomial alternative composition model predicted the number of children with trafficking indicators out of the number of rostered children aged 5 to 17 in the household, using the same alternative household composition variables. The child-level Koranic school sensitivity model added Koranic school or madrassa enrollment to the primary child-level model. The child-level alternative household composition model replaced household size with the number of children aged 5 to 17 and the number of non-child household members. “Not included” indicates that the predictor was not included in that model because it was either not defined at that level of analysis or was replaced by a corresponding household-level or child-level measure.
