# Supplementary material for: Household and child correlates of child trafficking indicators in Eastern Sierra Leone
Source: PLoS One. 2026 Oct 5;21(10):e0347898. doi: 10.1371/journal.pone.0347898 (PMC13637909; doi:10.1371/journal.pone.0347898)
Supplement: Supplementary file 6 [file pone.0347898.s006.docx]

**Household and Child Correlates of Child Trafficking Indicators in Eastern Sierra Leone**

**S6 Table. Random-intercept sensitivity models**

| Predictor | Household binary, EA random intercept OR [95% CI], p | Grouped-binomial household, EA random intercept OR [95% CI], p | Child-level, EA and household random intercepts OR [95% CI], p |
| --- | --- | --- | --- |
| Economic strain, standardized | 0.76 [0.66, 0.87], p < .001 | 0.75 [0.68, 0.83], p < .001 | 0.68 [0.58, 0.81], p < .001 |
| Material wealth, standardized | 1.22 [1.06, 1.39], p = .004 | 1.18 [1.08, 1.30], p < .001 | 1.43 [1.21, 1.68], p < .001 |
| Household size, standardized | 1.31 [1.18, 1.47], p < .001 | 0.94 [0.88, 1.00], p = .058 | 0.91 [0.80, 1.03], p = .142 |
| Any child not enrolled in formal school | 1.59 [1.24, 2.03], p < .001 | 1.37 [1.16, 1.61], p < .001 | Not included |
| Any orphan child in household | 1.59 [1.25, 2.02], p < .001 | 1.23 [1.05, 1.44], p = .009 | Not included |
| Child age, standardized | Not included | Not included | 3.47 [3.06, 3.94], p < .001 |
| Female child | Not included | Not included | 0.65 [0.54, 0.79], p < .001 |
| Child enrolled in formal school | Not included | Not included | 0.62 [0.46, 0.85], p = .003 |
| Orphan child | Not included | Not included | 1.46 [1.10, 1.95], p = .009 |
| Kailahun vs. Kenema | 1.25 [0.62, 2.53], p = .539 | 0.88 [0.44, 1.76], p = .712 | 0.80 [0.29, 2.20], p = .661 |
| Kono vs. Kenema | 1.20 [0.63, 2.29], p = .583 | 0.70 [0.38, 1.27], p = .240 | 0.91 [0.37, 2.25], p = .834 |

*Note.* OR = odds ratio; CI = confidence interval; EA = enumeration area. The household binary random-intercept model modeled whether a household had at least one rostered child aged 5 to 17 with one or more trafficking indicators and included an enumeration-area random intercept. The grouped-binomial household random-intercept model predicted the number of children with trafficking indicators out of the number of rostered children aged 5 to 17 in each household and included an enumeration-area random intercept. The child-level random-intercept model modeled whether each rostered child aged 5 to 17 had one or more trafficking indicators and included both enumeration-area and household random intercepts. All models included district fixed effects, with Kenema as the reference district. Continuous predictors were standardized. “Not included” indicates that the predictor was not included in that model because it was either not defined at that level of analysis or replaced by the corresponding household-level or child-level measure. Random-intercept models are presented as sensitivity analyses because the primary analyses used enumeration-area-clustered standard errors.
