# Supplementary material for: Household and child correlates of child trafficking indicators in Eastern Sierra Leone
Source: PLoS One. 2026 Oct 5;21(10):e0347898. doi: 10.1371/journal.pone.0347898 (PMC13637909; doi:10.1371/journal.pone.0347898)
Supplement: Supplementary file 7 [file pone.0347898.s007.docx]

**Household and Child Correlates of Child Trafficking Indicators in Eastern Sierra Leone**

**S7 Table. Random-intercept intraclass correlation summary**

| Model family | Model | N | Random effects included | EA ICC | Household ICC | Total cluster ICC | Convergence message |
| --- | --- | --- | --- | --- | --- | --- | --- |
| Household binary | Primary household model | 2,428 | EA random intercept | 0.402 | Not applicable | 0.402 | No convergence message |
| Household binary | Alternative household composition model | 2,427 | EA random intercept | 0.428 | Not applicable | 0.428 | No convergence message |
| Grouped-binomial household | Primary grouped-binomial model | 2,428 | EA random intercept | 0.441 | Not applicable | 0.441 | No convergence message |
| Child-level | Primary child-level model | 5,997 | EA and household random intercepts | 0.454 | 0.246 | 0.700 | No convergence message |
| Child-level | Alternative household composition model | 5,992 | EA and household random intercepts | 0.448 | 0.248 | 0.696 | No convergence message |

*Note.* EA = enumeration area; ICC = intraclass correlation coefficient. Household binary random-intercept models modeled whether a household had at least one rostered child aged 5 to 17 with one or more trafficking indicators. The grouped-binomial household random-intercept model predicted the number of children with trafficking indicators out of the number of rostered children aged 5 to 17 in each household. Child-level random-intercept models modeled whether each rostered child aged 5 to 17 had one or more trafficking indicators. Household-level models included enumeration-area random intercepts. Child-level models included both enumeration-area and household random intercepts. The total cluster ICC in child-level models is the sum of the enumeration-area and household ICCs. Random-intercept models are presented as sensitivity analyses to evaluate clustering; primary models used enumeration-area-clustered standard errors.
